# Establishment and initial implementation of the Australasian Pelvic Floor Procedure Registry

**DOI:** 10.1007/s00192-022-05435-8

**Published:** 2023-01-25

**Authors:** Randi T. Jayasinghe, Rasa Ruseckaite, Joanne Dean, Aruna Kartik, Anagi C. Wickremasinghe, Oliver Daly, Helen E. O’Connell, Amanda Craig, Anne Duggan, Dora Vasiliadis, Emmanuel Karantanis, Elizabeth Gallagher, Gwili Holme, James Keck, Jarrod Williams, Jennifer King, Jessica Yin, John Short, Kirstine Sketcher-Baker, Pip Brennan, Sally Rayner, Susannah Ahern

**Affiliations:** 1 Department of Epidemiology and Preventive Medicine, Monash University, Melbourne, Victoria 3004 Australia; 2 Department of Obstetrics and Gynaecology, Western Health, Melbourne, Victoria Australia; 3 Department of Surgery, University of Melbourne, Parkville, Australia; 4 Department of Health and Aged Care, Therapeutic Goods Administration, Canberra, Australia; 5 Australian Commission on Safety and Quality in Health Care, Sydney, Australia; 6 Consumer Representative, Australasian Pelvic Floor Procedure Registry, Melbourne, Australia; 7 St George Hospital, Kogarah, Australia; 8 Calvary John James, Canberra Private and Canberra Hospital, Canberra, Australia; 9 Commonwealth of Australia, Canberra, Australia; 10 St Vincent’s Private Hospital Melbourne, Melbourne, Australia; 11 Ministry of Health New Zealand, Wellington, New Zealand; 12 Westmead Hospital, Westmead, Australia; 13 Holywood Medical Centre (WA), Nedlands, Australia; 14 Christchurch Women’s Hospital & Southern Cross Hospital – Invercargill, Christchurch, New Zealand; 15 Clinical Excellence Queensland, Queensland Health, Herston, Australia

**Keywords:** Australasian Pelvic Floor Procedure Registry, Clinical Quality Registry, Pelvic Floor Procedures, Pelvic Organ Prolapse, Devices and Prostheses, Stress Urinary Incontinence

## Abstract

**Introduction and hypothesis:**

Stress urinary incontinence (SUI) and pelvic organ prolapse (POP) are common pelvic floor disorders (PFDs). Owing to significant adverse events associated with mesh-related pelvic floor procedures (PFPs) in a proportion of the surgically treated population, and deficits in collection and reporting of these events, the Australian Government identified an urgent need for a tracking mechanism to improve safety and quality of care. The Australasian Pelvic Floor Procedure Registry (APFPR) was recently established following the 2018 Senate Committee Inquiry with the aim of tracking outcomes of PFP involving the use of devices and/or prostheses, with the objective of improving the health outcomes of women who undergo these procedures. This paper will describe the APFPR’s aims, development, implementation and possible challenges on the way to its establishment.

**Methods:**

The APFPR has been developed and implemented in accordance with the national operating principles of clinical quality registries (CQRs). The minimum datasets (MDS) for the registry’s database have been developed using a modified Delphi process, and data are primarily being collected from participating surgeons. Patient recruitment is based on an opt-out approach or a waiver of consent. Patient-reported outcome measures (PROMs) providing additional health and outcome information will be obtained from participating women to support safety monitoring of mesh-related adverse events.

**Results:**

Currently in the Australasian Pelvic Floor Procedure Registry (APFPR) there are 32 sites from various jurisdictions across Australia, that have obtained relevant ethics and governance approvals to start patient recruitment and data collection as of January 2023. Additionally, there are two sites that are awaiting governance review and five sites that are having documentation compiled for submission. Seventeen sites have commenced patient registration and have entered data into the database. Thus far, we have 308 patients registered in the APFPR database. The registry also published its first status report and a consumer-friendly public report in 2022.

**Conclusions:**

The registry will act as a systematic tracking mechanism by collecting outcomes on PFP, especially those involving devices and/or prostheses to improve safety and quality of care.

## Introduction

Stress urinary incontinence (SUI) and pelvic organ prolapse (POP) are common pelvic floor disorders (PFDs) with prevalence increasing with parity and age. In Australia, up to 50% of women are affected by SUI and POP [1, 2] with a 20% lifetime risk of receiving a pelvic floor reconstructive procedure [3]. It is estimated that by 2030, there will be a 34% increase in the number of women affected by PFDs [4].

For an uncertain number of Australian women, there has been significant morbidity associated with complications related to pelvic floor mesh [5, 6]. The 2018 Senate Committee Inquiry into the “Number of women in Australia who have had transvaginal mesh implants and related matters” highlighted the inadequacy of current reporting systems in estimating the number and outcomes of pelvic floor procedures (PFPs) being undertaken in Australia. The inquiry emphasised the lack of systematic tracking of these procedures in the short and long term with respect to quality, safety and relative effectiveness in the face of significant variation in clinical outcomes [7].

Clinical quality registries (CQRs) are organisations that systematically monitor the quality of health care within specific clinical domains by routinely collecting, analysing and reporting health-related information [8]. Where they have been introduced at a state or national level, registries have become one of the most clinically valued tools for quality improvement [8, 9]. Registries can improve safety and quality of care by providing credible risk-adjusted data, providing an early warning if the safety or quality of care deteriorates, giving clinicians information about how their outcomes benchmark with others, both locally and where appropriate internationally; and identifying and investigating variations in clinical practice and outcomes.

Following the recommendations of the 2018 Senate Inquiry into transvaginal mesh and related matters, the Commonwealth Department of Health provided initial 3-year funding to establish a CQR to monitor the safety and outcomes of PFPs undertaken in Australia [10]. The Australasian Pelvic Floor Procedure Registry (APFPR) was established in 2019 to address systemic deficits in the collection, analysis and reporting of PFPs, to establish early warning systems, and to provide feedback to clinicians, hospitals, regulatory bodies and ultimately the public regarding the status of pelvic floor interventions [5, 10].

This project is aimed at developing a longitudinal population-based CQR to optimise the safety and quality of care provided in individuals undergoing SUI- and POP-related PFPs. This will be achieved by tracking and monitoring efficacy and adverse outcomes of devices and/or prostheses, and identifying variability in clinical outcomes amongst such individuals. The APFPR is also responding to the recommendations of the Australian Commission on Safety and Quality in Health Care (ACSQHC) that emphasise postoperative monitoring and collection of patient-reported outcome measures (PROMs) [11]. This paper describes the establishment and initial implementation of the APFPR.

## Materials and methods

The APFPR was commissioned with the aim of developing a systematic tracking mechanism to monitor care and safety outcomes through the collection of high-quality epidemiological data on PFP, especially those involving devices and/or prostheses, undertaken in Australian health care institutions.

### Consumer involvement

The APFPR is committed to facilitating meaningful consumer consultation and engagement to inform the registry. To ensure that our decision-making and advisory bodies are informed by consumer-focused views, formal consumer representatives and other forms of consumer engagement are being fostered and will be supported as the registry matures. The consumer representatives will continue to contribute to the registry design, review the instruments, assist with development of the PROMs and informational material. The representatives will also continue to review the findings and contribute to the dissemination plan, thereby raising consumer awareness influencing public support for the APFPR.

### Governance structure

#### Coordinating centre

The APFPR coordinating centre, located at the Department of Epidemiology and Preventive Medicine, Monash University, manages the core registry activities with oversight by Steering and Management committees that meet every quarter.

#### Steering committee

The Steering Committee (SC) provides strategic direction, policy deliberation and monitors the progress of the Registry. Membership includes consumer representatives and clinicians from relevant specialist groups such as urogynaecologists, gynaecologists and urologists. The APFPR SC currently includes two consumer representatives, one of whom has lived experience, and another who is an experienced consumer advocate. Representatives of the Commonwealth Government and other jurisdictions, the Therapeutic Goods Administration (TGA), the ACSQHC, as well as a non-Monash device registry staff member.

#### Management committee

The Management Committee (MC), with specialist groups and University representatives, provides additional input into clinical issues as representatives of their various societies.

### Ethics review

The National Health and Medical Research Council (NHMRC) stipulates that all medical registries and quality assurance (QA) projects require Ethics committee review [12]. The APFPR received Ethics approval under the National Mutual Acceptance (NMA) scheme from the Monash Health Human Research Ethics Committee (HREC) in 2020 (Project ID: RES-20-000-444A) [13]. Under the NMA scheme, every participating surgeon/site in a multi-centre research project will require site-specific authorisation before any registry activity can commence at the individual sites.

### Surgeon engagement

Clinicians, SC and MC representatives assist in identifying high-volume sites and Principal Investigators (PIs) for each site. PFPs are undertaken by gynaecologists, urogynaecologists and urologists, at both metropolitan and regional and in public or private hospital settings in Australia. Opportunities to promote the registry at hospital events or forums that are attended by surgeons from relevant specialist groups, heads of departments, clinical leads, and directors are leveraged to encourage participation. Public hospitals are enrolled as one unit, with the PI being the site contact person. Each surgeon receives individualised access to the database upon signing a database access request. Private surgeons are recruited individually after execution of a clinician-level agreement before they are enrolled and provided with database access. All clinicians receive training on patient eligibility and data entry. They are provided with an introductory pack with relevant forms, flyers and material necessary for patient registration.

To recognise the contribution of clinicians, the APFPR is liaising with the professional colleges to facilitate the awarding of Continuing Professional Development (CPD) points to surgeons participating in the registry. Additionally, the APFPR database allows surgeons to run and filter reports of their own patients in real time, and will provide site and surgeon level reporting when sufficient recruitment volumes are reached.

### Registry design

The APFPR is a prospective, longitudinal multicentred project aimed at capturing the entire number of SUI and POP procedures in women involving devices and/or prostheses; this is being rolled out in a modular fashion. The first module focuses on SUI-related mesh implantation, revision or removal. The second module focuses on POP-related mesh implantation, revision or removal.

### Registry population

All female patients undergoing a SUI and/or POP procedure with a participating surgeon/site are eligible to participate in the registry.

### Recruitment model

#### Opt-out process

The APFPR uses an opt-out consent model (14-day opt-out period) to recruit patients into the registry. A waiver of consent has been approved for those who do not have the cognitive capacity to consent to being recruited into the registry. Opt-outs and waivers enable population coverage to be maximised, which is important in identifying individual device performance.

#### Participant recruitment

**Fig. 1:**
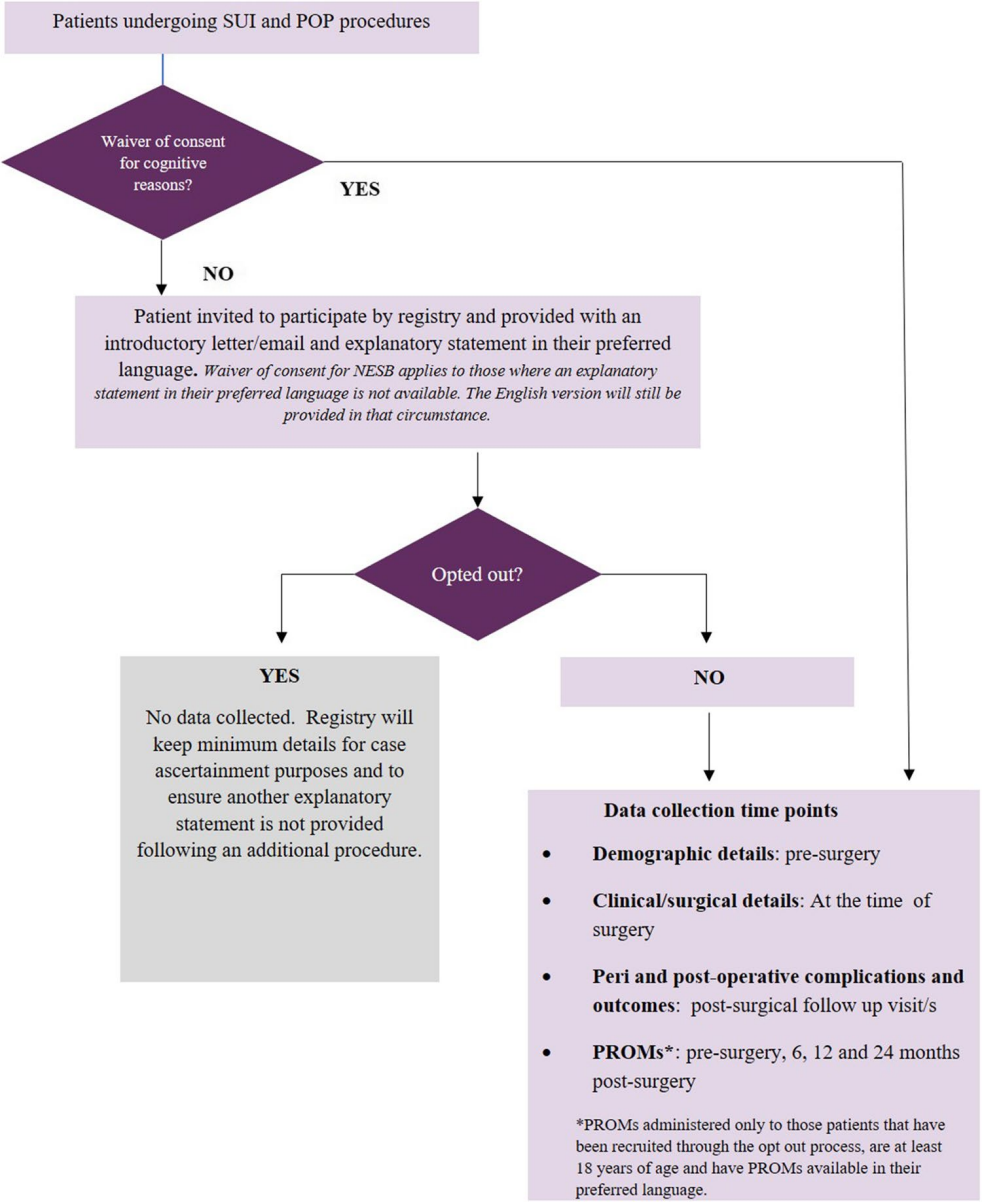
Australasian Pelvic Floor Procedure Registry (APFPR) registry recruitment and data collection schema. *SUI* stress urinary incontinence, *POP* pelvic organ prolapse, *NESB* non-English-peaking background, *PROMs* patient-reported outcome measures

Patient recruitment is a shared responsibility between the APFPR and the participating surgeon. The surgeon provides an introductory leaflet to the patient at a pre-surgical consultation and registers the patient’s demographic details into the database. The APFPR Coordinating team then dispatches the participant’s explanatory statement to the patient or to the parent/guardian (if the patient is under 18 years of age), which clearly outlines the opt-out process. An outline of the recruitment framework is provided in Fig. 1.

Once the opt-out period of 14 days has elapsed and the invited patient does not opt out, the clinical and surgery forms on the database become functional and can be used by the surgeon to add data. Identifiable clinical, diagnostic, surgical and post-operative data about PFPs will be entered into the registry database by the participating surgeon or delegate.

#### Data collection and minimum dataset

The APFPR collects information from participating sites and registry participants. Data collected at baseline and post-surgery from the participating sites comprise an evidence-based minimum dataset (MDS) of standardised data items to ensure that the data collected are consistent, providing reliable and comparable data for analysis. Collected data items are broadly categorised in Table 1 with the relevant time points for collection, including demographic, comorbidity, surgical, device and complication details. PROMs administration is currently being piloted and will be published separately.

**Table 1:** Registry data collection items and timetable

	Data items collected	Presurgical consultation	At the time of surgery	Post-surgical follow-up (6 weeks)	6 months	Responsibility
APFPR data collection	Name, DOB, address, phone number, email, date of and type of procedure^a^, Medicare number, ATSI status	X				Surgeon/site
	Eligibility confirmation and consent	X				Surgeon/site and registry
	Procedure details; surgery date and procedure details; pelvic floor status, comorbidities, mesh/prosthetic device details, intrasurgical complications		X			Surgeon/site
	Post-surgical follow-up; surgical outcomes, complications; return to theatre and readmission details; health status			X	X	Surgeon/site
	QoL questionnaire/PROMs^b^	Piloting implementation currently; PROMs administration will be published separately				Registry

*APFPR* Australasian Pelvic Floor Procedure Registry, *DOB* date of birth, *ATSI* Aboriginal or Torres Strait Islander status, *QoL* quality of life, *PROMs* patient-reported outcome measures

^a^Guardian contact details will also be collected for those under 18 years of age

^b^PROMs are not administered to minors, NESB without a translated explanatory statement and PROMs, or those with cognitive difficulties

A Delphi process has been used to develop an MDS for the SUI and POP modules in the registry’s database [14–16]. A detailed methodology of this Delphi process will be published separately. This will account for procedure type, outcomes and risk factor adjustments and categorised into several domains: patient demographics, surgical data, complications and adverse outcomes, as well as validated PROMs.

#### Data management

The APFPR uses a secure web-based platform (REDCap) to enter and manage data [17, 18]. Study data are collected and managed using REDCap electronic data capture tools hosted at Monash University. Data collected through REDCap is directly and permanently stored on infrastructure located in Australia. Each registry database user will have their own username and password to access the database.

Data completeness and accuracy are optimised through in-built validation and completion checks in the registry database to minimise data entry error, where sites/surgeons enter data directly into the database (such as data entry controlled by form logic, date restrictions and in-built limitations to feasible data only, the use of built-in edit checks to ensure that data meet required formats and ranges, validation rules applied at the time of submission with alerts to assist with errors and missing data). Additional quality checks post-data entry include checks for duplicate data, including duplicate patients, cross-checking data obtained from multiple sources—surgical notes vs self-report, missing data and data consistency. Quality checks are undertaken by APFPR registry staff for case ascertainment, data accuracy and completion at regular intervals. Missing or erroneous data, when identified, are referred back to contributing sites or surgeons for review.

#### Data access policy

Researchers and medical professionals working at research institutions, hospitals, private entities, government or other health services within Australia and industry are eligible to request access to data held within the registry. The APFPR will only release the least sensitive level of data that is practicable to fulfil the uses identified in the research proposal submitted with the data request. Tabulated aggregate data with a cell count of less than five will be suppressed to prevent re-identification of an individual patient, physician or organisation.

Any application for data will be reviewed in accordance with the APFPR data access policy and will require APFPR SC approval as well as low-risk ethics approvals where required. Patient requests for data access to their information will be approved by the APFPR SC. Patients will need to provide identifying information before the data release. The APFPR Coordinating Centre will be the point of contact for matters relating to access to registry data.

#### Data analysis and reporting

Initial data analysis for the APFPR will focus on descriptive analysis to provide a summary of patient characteristics, clinical quality indicators (CQIs) and PROMs when available. This will be reported using continuous variables summarised using the median, interquartile range, mean and standard deviation and for categorical variables counts and proportions. These data will be included in reports to sites or surgeons and annual public reports. Adverse events and complications associated with surgery, including the number of revisions and explanations reported by surgeons and/or patients, will be used to identify variation in the number, type and severity of events. This information will be used to determine safety concerns related to mesh and other factors. Additionally, performance metrics analyses will include opt-out rates, recruitments of sites and response rates for PROMs. Risk-adjusted funnel plots will compare the quality of care using CQIs across sites.

#### Current status

Currently, there are 32 sites from various jurisdictions across Australia that have obtained relevant ethics and governance approvals to start patient recruitment and data collection as of January 2023. Additionally, there are two sites that are awaiting governance review and 5 sites that are having documentation compiled for submission. Seventeen sites have commenced patient registration and have entered data into the database. Thus far, we have 308 patients registered into the APFPR database.

#### Research output and communication

The APFPR provides information updates to its stakeholders through a website and a quarterly communiqué issued to the colleges, clinicians, nursing staff, consumer groups and hospital departments. Our first status report was published in October 2022, followed by a consumer-friendly public report in November 2022. Once the dataset is mature, the APFPR will produce annual reports with de-identified, aggregate data and site/clinician reports that will serve to furnish benchmarking information on outcomes.

## Discussion

Stress urinary incontinence and POP are identified as two of the most debilitating illnesses that increase with parity and age in females. It is estimated that the incidence of women with at least one pelvic floor disorder will double by 2050 and that the lifetime risk of undergoing an SUI or a POP procedure is 20% [2, 3]. It has been found that nulliparous women experience SUI four times more often than men, and the prevalence further increases after the first pregnancy (37.4%) [1]. The overall prevalence of any incontinence reported in women was also 35.3% [1]. Overall, findings to date elicit that women’s lack of awareness and knowledge regarding these gynaecological conditions leads to limited health care-seeking, which contributes to a greater impairment in quality of life.

The APFPR monitors the diagnosis, treatment and outcomes of SUI and POP pelvic mesh procedures, undertaken by participating clinicians in Australia and New Zealand. The APFPR is designed to systematically detect adverse outcomes over time related to the use of devices and/or prostheses, provide this feedback to clinicians and the national medical regulator, and to thereby improve the quality of care for patients undergoing PFPs.

The APFPR has committed representation from the multiple clinical craft groups and their associated professional colleges; from consumer representatives; jurisdictional level support; and Commonwealth funding and support. The APFPR, through the establishment of systematic data collection, will also empower patients to make informed decisions about care and treatment options. The utilisation of opt-out and waivers of consent in the recruitment approach allow coverage and generalisability to be maximised, while at the same time preserving patient autonomy in keeping with the principles of Good Research Practice.

The registry will promote public confidence that PFPs with mesh, other devices and/or prostheses are performed under the oversight of a newly introduced robust quality assurance programme; as highlighted in the Senate Inquiry, tracking of complications associated with the procedures, both short-term and long-term, will be used to determine safety concerns related to mesh and other factors. The APFPR will also contribute to TGA post-marketing surveillance, which is currently dependent on public reporting, the latter known to be under-utilised by consumers and clinicians.

The International Continence Society–International Urogynaecological Association Mesh Complication Classification Scale [19, 20] is being used to capture complications, this being one of the first registries to do so. Additionally, capturing PROMs and proposed data linkage to State or Commonwealth datasets will provide a more comprehensive perspective and holistic picture of patient outcomes and quality of life. The APFPR will also track devices and their outcomes and routinely provide device performance reports to the TGA using de-identified aggregated data. In the event of a hazard alert involving mesh or other prosthetic products, the surgeons or sites will be able to immediately identify their own patients as having had the product in question.

The APFPR is not without its challenges. The registry relies on surgeon participation over and above their regular clinical duties. Although CQR will provide data with high external validity, there is a risk of bias regarding providers choosing patients for the registry. The APFPR will conduct sample audits and undertake case ascertainment to monitor data quality.

Case ascertainment and sample audit that will be performed by the registry will minimise the risk of bias regarding patient and surgeon participation. This will involve auditing contributing hospitals and comparing site numbers (patients, procedures etc.) against registry numbers. The cross checking of data will allow for the registry to identify any data that have not been captured by the APFPR.

The APFPR was initially conceived as an initiative to primarily monitor mesh use; the continued regulatory restrictions, namely the ban on transvaginal mesh for POP procedures as well as single-incision mini-slings for SUI and negative consumer sentiment have resulted in a marked reduction of mesh PFPs being performed in Australia [7, 21]. As a result, there has been a reduction in overall PFPs being undertaken for both SUI and POP during this time, although an increase in bulking agent injections and abdominal procedures is being observed [22]. The APFPR is reviewing comparable international registries to ensure that it remains adaptable and flexible to respond to changing practices. Although a number of international registries also include native tissue (autologous) procedures, these have been generally considered safe and effective within their patient population, and may not warrant oversight from a national registry.

Most international registries/databases capture data on mesh-related and native tissue procedures in order to provide feedback on comparative outcomes. The British Society of Urogynaecology’s audit database in its second national report on SUI surgery finds success rates varying from 89% for native tissue procedures to 56% for bulking agents and a complication rate of up to 5% [23]. The marked decline in mesh availability is driving research/innovation on alternative products for use and the APFPR is well placed to provide the ideal foundation for testing new techniques/products and gathering evidence on long-term outcomes for the foreseeable future [24]. The registry will serve as a data spine for new research into pelvic floor treatments.

### Strengths and limitations

This paper outlines the establishment of the APFPR for tracking and monitoring PFPs performed in Australia and New Zealand, including the ethics, governance, consumer and clinical engagement, minimum dataset, data collection, data access policy and reporting. Addressing the recommendations of the Senate Inquiry and ACSQHC, it is the first registry to be established in Australia and New Zealand to routinely track and monitor PFPs and outcomes of patients following those procedures, specifically those involving devices and/or prostheses. The implementation of PROMs will also add to the existing knowledge on pelvic floor disorder-related issues faced by women and support the monitoring of mesh-related devices in the long term.

### Future directions

Given the need for safe and high-quality treatments for PFDs, the APFPR can serve as a foundation to evaluate new prostheses and treatments available in the future for PFPs. Implementation of PROMs will also enable additional information and support safety monitoring of mesh-related adverse events in the long term.

## Conclusion

The APFPR is a CQR for women undergoing SUI and/or POP procedures involving a prostheses, mesh and other devices in Australia and New Zealand. It will be an effective systematic tracking mechanism that will contribute to improving the quality of care and patient outcomes for this population.
